# Supplementary material for: Preliminary placement and new records of an overlooked Amazonian tree, Christiana mennegae (Malvaceae)
Source: PeerJ. 2021 Nov 15;9:e12244. doi: 10.7717/peerj.12244 (PMC8601058; doi:10.7717/peerj.12244)
Supplement: Supplemental Information 1 [file peerj-09-12244-s001.docx]

Electronic Supplementary Material

**Preliminary placement and new records of an overlooked ee, *Christiana mennegae* (Malvaceae)**

Rafael Gomes Barbosa-Silva, Thales Silva Coutinho, Santelmo Vasconcelos, Delmo Fonseca da Silva, Guilherme Oliveira, Daniela Zappi

Corresponding author: Rafael G. Barbosa-Silva

Email: [rafa.g29@gmail.com](mailto:rafa.g29@gmail.com)

### **Material and methods**

***DNA Extraction and Phylogenetic analysis.***

Young leaf tissues of *C. mennegae* were collected in 2% CTAB-NaCl saturated buffer (Rogstad, 1992), then the automated DNA extraction protocol with QIAcube HT (Qiagen) was followed using the ‘Q protocol V1’ of the QIAamp 96 DNA Kit (Qiagen), according to manufacturer’s instructions. The PCR conditions followed Ramalho et al. (2018) and the bidirectional sequencing reactions were performed in an ABI 3730 DNA Analyzer (Applied Biosystems), with the BigDye Terminator v3.1 Cycle Sequencing Kit (Thermo Fisher). Forward and reverse reads obtained for each marker were assembled in a consensus sequence with PIPEBAR (Oliveira et al., 2018), using the recommended settings for coding (*rbc*L) and intergenic (ITS2) regions.

Markov Chain Monte Carlo (MCMC) analyses were run on MrBayes for 50,000,000 generations, starting with a random tree and sampling every 5,000 generations. The first 25% of the trees were discarded as the burn-in, and the remaining trees were used to generate a majority-rule consensus tree. Clades with posterior probability values (PP) ≥ 0.95 were considered as well-supported (Eriksson, 2001; Alfaro, 2003; Kolaczkowski & Thornton, 2007). ML tree searches and bootstrap estimates of clade support were conducted with RAxML (Stamatakis et al., 2005), using the GTR substitution model with gamma-distributed rate heterogeneity among sites and the proportions of invariable sites estimated from the data. For the ML analyses, clades presenting bootstrap support values (BS) ≥ 75% were considered as strongly supported (Hillis & Bull, 1993). Both BI and ML analyses were performed at the CIPRES Science Gateway (Miller et al., 2010).

**Results**

Table

Table 1. Morphological comparison among species of *Christiana* DC. (based on our own data and Jansen-Jacobs & Westra 1983; Kubitzki 1995; Sprague 1908; Toledo 1952; and Tschá et al. 2002).

|  | ***C. africana*** | ***C. eburnea*** | ***C. macrodon*** | ***C. menneage*** | ***C. vescoana*** |
| --- | --- | --- | --- | --- | --- |
| Leaf-blade margins | Entire | Entire | Sparsely dentate | Entire or rarely slightly erose | Entire |
| Leaf-blade shape | Widely ovate | Ovate | Widely ovate to circular | Narrowly elliptic, lanceolate or oblanceolate | Widely ovate |
| Leaf-blade base | Cordate | Truncate to subcordate | Cordate | Rounded to obtuse, rarely cordate | Cordate |
| Trichomes type on the leaf-blade | Stellate | Stellate | Stellate | Stellate and glandular | Stellate |
| Fruit shape | Depressed-globose | Subturbinate to turbinate | Transversely ellipsoid | Depressed-globose | Depressed-globose |
| Fusion of the fruits | Apocarpous | Syncarpous | Syncarpous | Syncarpous | Syncarpous |

### **Micromorphology study**

The glandular trichomes type is found both on petioles and sparsely distributed on the lower surface of the leaf-blade (Fig. 3 A–C). Both simple and two-armed trichomes were observed only on petioles (Fig. 3 A), whereas stellate-rotate are observed in petiole and vascularization on abaxial face (Fig. 3 A andC). Stellate-multiangulate trichomes were observed only on reproductive structures, on the external surface of the calyx and fruit (Fig. 2 F, Fig 3 D-F). It is important to note that while the trichomes are sparse in most parts of the plants, they are concentrated only in the reproductive structures (Fig. 3 D-F).

### **Phylogenetic relationships**

The figure 1-2 below show two tree topology of Maximum Likelihood (Fig.1) and Bayesian Inference (Fig. 2).

**
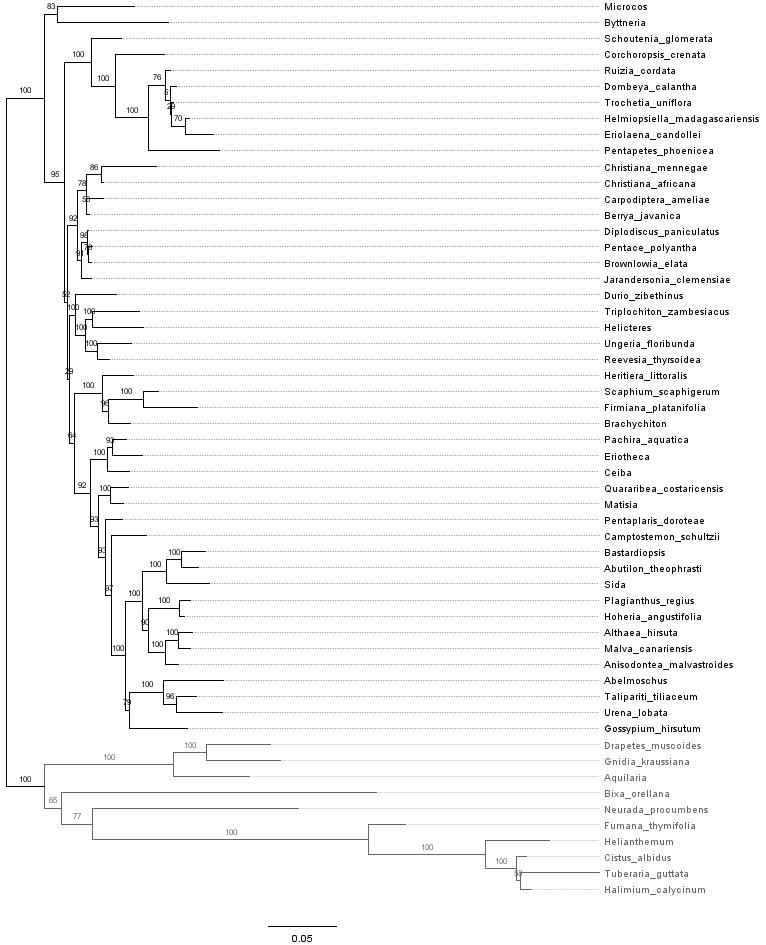
**

Figure 1. Resulting from a Maximum Likelihood analysis of the combined dataset (*rbc*L, *atp*B, *trn*K-*mat*K, *ndh*F and ITS2).

**
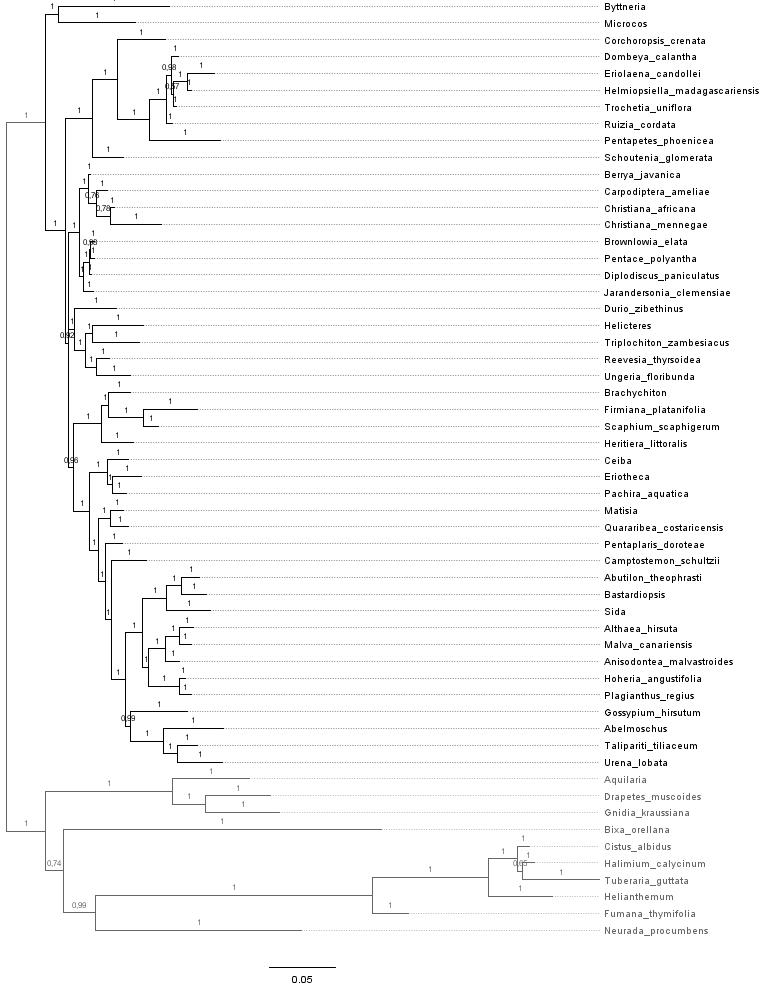
**

Figure 2. Resulting from a Bayesian analysis of the combined dataset (*rbc*L, *atp*B, *trn*K-*mat*K, *ndh*F and ITS2).

**References**

Alfaro ME. 2003. Bayes or Bootstrap? A Simulation Study Comparing the Performance of Bayesian Markov Chain Monte Carlo Sampling and Bootstrapping in Assessing Phylogenetic Confidence. *Molecular Biology and Evolution* 20:255–266. DOI: 10.1093/molbev/msg028.

Eriksson T. 2001. *Autodecay, version 5.0. Software program distributed by the author.* Sweden: Bergius Foundation, Royal Swedish Academy of Sciences.

Hillis DM, Bull JJ. 1993. An empirical test of bootstrapping as a method for assessing confidence in phylogenetic analysis. *Systematic Biology* 42:11.

Jansen-Jacobs MJ., Westra LYT. 1983. Studies on the flora of the Guianas. II. A new species of Asterophorum (Tiliaceae) from Suriname. :377–383.

Kolaczkowski B, Thornton JW. 2007. Effects of branch length uncertainty on Bayesian posterior probabilities for phylogenetic hypotheses. *Molecular Biology and Evolution* 24:2108–2118. DOI: 10.1093/molbev/msm141.

Kubitzki K. 1995. Asterophorum and Tahitia congeneric with Christiana (Tiliaceae).

*Bot. Jahrb. Syst. 116(4): 537-542.*

Miller MA, Pfeiffer W, Schwartz T. 2010. Creating the CIPRES Science Gateway for inference of large phylogenetic trees. In: *2010 Gateway Computing Environments Workshop (GCE)*. New Orleans, LA, USA: IEEE, 1–8. DOI: 10.1109/GCE.2010.5676129.

Oliveira RRM, Nunes GL, de Lima TGL, Oliveira G, Alves R. 2018. PIPEBAR and OverlapPER: tools for a fast and accurate DNA barcoding analysis and paired-end assembly. *BMC Bioinformatics* 19. DOI: 10.1186/s12859-018-2307-y.

Ramalho AJ, Zappi DC, Nunes GL, Watanabe MTC, Vasconcelos S, Dias MC, Jaffé R, Prous X, Giannini TC, Oliveira G, Giulietti AM. 2018. Blind testing: DNA barcoding sheds light upon the identity of plant fragments as a subsidy for cave conservation. *Frontiers in Plant Science* doi: 10.3389/fpls.2018.01052:1–10. DOI: doi: 10.3389/fpls.2018.01052.

Rogstad SH. 1992. Saturated NaCl-CTAB solution as a means of field preservation of leaves for DNA analyses. *Taxon* 41:701–708.

Sprague TA. 1908. Decades Kewenses. Plantarum Novarum in Herbario Horti Regii Conservatarum. Decas L. *Bulletin of Miscellaneous Information (Royal Gardens, Kew)* 1908:249–255. DOI: 10.2307/4111573.

Stamatakis A, Ludwig T, Meier H. 2005. RAxML-III: a fast program for maximum likelihood-based inference of large phylogenetic trees. *Bioinformatics* 21:456–463. DOI: 10.1093/bioinformatics/bti191.

Toledo JF. 1952. Notulae de aliquot plantis brasiliensibus novis vel minus cognitis. *Arquivos de botânica do estado de São Paulo* 3:29–30.

Tschá MC, Sales MF, Esteves GL. 2002. Tiliaceae Juss. no estado de Pernambuco. *Hoehnea* 29:1–18.
